# Hyperosmolarity Impairs Human Extravillous Trophoblast Differentiation by Caveolae Internalization

**DOI:** 10.3389/fphys.2021.760163

**Published:** 2021-12-06

**Authors:** Julieta Reppetti, Yollyseth Medina, Mariana Farina, Alicia E. Damiano, Nora Alicia Martínez

**Affiliations:** ^1^Laboratorio de Biología de la Reproducción, Instituto de Fisiología y Biofísica Bernardo Houssay (IFIBIO) - CONICET- Facultad de Medicina, Universidad de Buenos Aires, Buenos Aires, Argentina; ^2^Laboratorio de Fisiopatología Placentaria, Centro de Estudios Farmacológicos y Botánicos (CEFYBO) - CONICET, Facultad de Medicina, Universidad de Buenos Aires, Buenos Aires, Argentina; ^3^Cátedra de Biología Celular y Molecular, Departamento de Ciencias Biológicas, Facultad de Farmacia y Bioquímica, Universidad de Buenos Aires, Buenos Aires, Argentina

**Keywords:** human extravillous trophoblast, hyperosmolarity, caveolae/Cav-1, cell migration, endovascular differentiation

## Abstract

We recently reported that an intact caveolar structure is necessary for adequate cell migration and tubulogenesis of the human extravillous trophoblast (EVT) cells. Emerging evidence supports that hyperosmolarity induces the internalization of caveolae into the cytoplasm and accelerates their turnover. Furthermore, signaling pathways associated with the regulation of trophoblast differentiation are localized in caveolae. We hypothesized that hyperosmolarity impairs EVT differentiation and caveolae/caveolin−1 (Cav-1) participates in this process. EVT cells (Swan 71 cell line) were cultured in complete Dulbecco’s Modified Eagle Medium/Nutrient Mixture F-12 and exposed to hyperosmolar condition (generated by the addition of 100 mM sucrose). Hyperosmolarity altered the EVT cell migration and the formation of tube-like structures. In addition, cell invasion was decreased along with a reduction in the latent and active forms of matrix metalloproteinase-2 (MMP−2) secreted by these cells. With respect to Cav-1 protein abundance, we found that hyperosmolarity enhanced its degradation by the lysosomal pathway. Accordingly, in the hyperosmolar condition, we also observed a significant increase in the number of vacuoles and the internalization of the caveolae into the cytoplasm. Taken together, our findings suggest that hyperosmolarity may induce caveolae internalization and increase their turnover, compromising the normal differentiation of EVT cells.

## Introduction

The success of pregnancy depends on the normal placental development that involves the differentiation of the highly proliferative and undifferentiated trophoblasts into two different general pathways. On one hand, the mononucleated cytotrophoblasts (CTBs) fuse into multinucleated syncytiotrophoblasts (STBs) to establish the placental floating villous, which is involved in the fetal-maternal transport and hormones synthesis. On the other hand, a pool of CTB cells proliferates forming the trophoblast column to establish the anchoring villi that attach the placenta to the uterine wall (Bilban et al., 2010). A group of cells that acquire an invasive phenotype detaches from these structures, loses their polarity, and differentiates into interstitial extravillous trophoblasts (inEVTs), which can migrate and invade the decidua and myometrium. Continuing on the path of differentiation, these inEVTs can become endovascular EVTs (enEVTs), which remodel the spiral uterine arteries by disruption of endothelium–myometrium interaction and replacing the endothelial cells, acquiring an “endothelial-like phenotype” (Bischof and Campana, 1996; Cartwright et al., 2010; Cartwright and Whitley, 2017).

Although these events are not entirely clear, it is known that they are tightly regulated in a spatiotemporal manner. Different works show a switch in the adhesion molecule profile and the production of proteases suggesting that these processes must be regulated by several signals (Fisher et al., 1989; Haider et al., 2018). Consequently, defects in these highly-coordinated processes in the early first trimester of gestation can lead to pathologies associated with placental insufficiency as fetal death, fetal growth restriction (FGR), and preeclampsia (PE) (Cartwright et al., 2010; Ji et al., 2013).

Previous findings have shown that these gestational pathologies are associated with a hemoconcentration state with reduced plasma volumes, which lead to hyperosmolar stress (Huisman and Aarnoudse, 1986; Mello et al., 2002).

Several studies aim to elucidate the molecular mechanisms that underlie the differentiation of the trophoblast into the endovascular phenotype. Nevertheless, little is known about the effects of alterations in the trophoblast microenvironment during early placental development on its capacity for differentiation and vascular remodeling.

Zhong et al. (2007) observed in mouse placental stem cells and mouse embryos that hyperosmolar stress induces cell death by apoptosis in a dose- and time-dependent manner. According to these results, we recently reported in EVT cells that osmolarities greater than 450 mOsM induce apoptosis by a mechanism that involves the activation of the transient receptor potential vanilloid-1 (TRPV-1) channel. In these situations, hyperosmolarity acts like a lethal stressor (Reppetti et al., 2019).

Two gestational pathologies can be mentioned with respect to clinical situations in which a failure in controlling osmolarity is observed. The most severe of them is uncontrolled gestational diabetes, which corresponds to lethal hyperosmolarity and leads to spontaneous abortions (Gonzalez et al., 2007). The other one is preeclampsia, which occurs with placental insufficiency and is associated with sublethal hyperosmolarity (Xiong et al., 2009). Therefore, the origin of placental dysfunction may be an abnormal response of the trophoblast to sublethal hyperosmolar stress.

Until now, there are no reports on the effect of sublethal hyperosmolar stress on enEVT differentiation.

Many of the signaling pathways involved in trophoblast differentiation have receptors located in caveolae (Liu et al., 1996; Song et al., 1996; Moreau et al., 2002; Daoud et al., 2006; Xie et al., 2011). The caveolae are small specialized plasma membrane microdomains with a diameter of 60–80 nm. Caveolin-1 (Cav-1) is the major integral membrane protein required for the formation of these structures. These plasma membrane microdomains are a scaffolding platform that integrates many different signaling pathways (Head et al., 2008; Patel et al., 2008). Several reports have demonstrated that caveolae and Cav-1 participate in the migration and invasion of malignant cells (Carver and Schnitzer, 2003; Grande-García and del Pozo, 2008). In the human placenta, we have recently reported that an intact caveolar structure is necessary for adequate cell migration and endovascular differentiation of the EVT cells (Reppetti et al., 2020). Nevertheless, the association among hyperosmotic microenvironment, enEVT differentiation, and caveolae was not explored yet.

Therefore, we hypothesized that sublethal hyperosmolarity alters the normal structure of the caveola/Cav-1 leading to abnormal enEVT differentiation. Our main aim was to evaluate the effects of hyperosmolarity on all the processes related to endovascular-EVT differentiation such as cell mobility, cell invasion, and the formation of tube-like structures. Then, we studied if the hyperosmolarity affects the caveola/Cav-1 structure.

## Materials and Methods

### Cell Culture

Swan 71 cell line, obtained from CTBs isolated from a 7−week placenta, was used (Straszewski-Chavez et al., 2009). Cells were cultured in Dulbecco’s Modified Eagle Medium/Nutrient Mixture F-12 (DMEM/F-12, Life Technologies Incorporation BLR, Grand Island, NY, United States) supplemented with 10% fetal bovine serum (FBS) (Laboratorio Natocor, Cordoba, Argentina), 100 U/ml penicillin, 100 μg/ml streptomycin, 0.25 μg/ml amphotericin B, and 5 mM L-glutamine (Invitrogen, Carlsbad, CA, United States) up to confluence. Cells were cultured at 37°C and 5% carbon dioxide (CO_2_).

At confluence, cells were arrested overnight (standard medium supplemented with 0.5% FBS). Then, a 100 mM sucrose solution was added to the arrest medium to generate the hyperosmolar condition or vehicle [phosphate-buffered saline (PBS)] was added to the arrest medium to generate the iso-osmolar condition (control) and incubation was continued for 24 h. Osmolarity was measured using a pressure vapor osmometer VAPRO™ (Wescor Incorporation, Logan, UT, United States) as described previously (Reppetti et al., 2019).

Additionally, for Western blot experiments, Swan 71 cells were treated for 6 h with 10 μM MG132 (Calbiochem, San Diego, CA, United States), a proteasomal inhibitor, and 10 mM ammonium chloride (NH_4_Cl) (Biopack, Buenos Aires, Argentina) used as a lysosomal inhibitor.

### Cell Viability Assay

Cell viability was assessed by the 3-(4,5-dimethylthiazol-2-yl)-2,5-diphenyl tetrazolium bromide (MTT) (Sigma-Aldrich Corporation, San Luis, Mosby, MO, United States) assay as described previously (Reppetti et al., 2019). Swan 71 cells (7 × 10^3^ per well) were loaded in a 96-well microplate until confluency in DMEM/F-12 with 10% FBS. Then, the treatments were performed and cells were arrested in DMEM/F-12 0.5% for 24 h. After treatments, the medium was removed and 100 μl of MTT (0.5 mg/ml in PBS) was added and incubated for 2 h at 37°C. Once the incubation time has ended, the formazan crystals were solubilized in 100 μl of absolute ethanol and the optical density was measured at 570 nm in an ELISA reader. The cell viability was expressed as arbitrary densitometric units.

### Wound Healing Assay

After treatments, the confluent monolayer of hyperosmolarity-treated cells and control cells were scratched with a 200-μl pipette tip across the center of the wells to produce a wound. Wounded monolayers were examined at 0, 6, and 24 h and photographed using an inverted microscope (Nikon, Eclipse E:200) equipped with a camera. The ImageJ 1.51r^®^ software package (Bethesda, MD, United States) was used to quantify the cell migration rate. On one hand, we calculated the wound area for each photograph and expressed the migration rate as the percentage of area reduction or wound healing. The scratch distance at 0 h was considered as 0% of wound healing. We also measured the width of the wound as the average distance between the edges of the scratch of each photograph (measured in pixels) and with this value, we calculated the migration speed at each time, dividing the change in the width of the wound by the time spent on each migration (Grada et al., 2017).

### Gelatin Zymography

The activities of metalloproteinases (MMPs) were determined by gelatin zymography assay as described previously (Reppetti et al., 2020). Culture medium from control or hyperosmolarity-treated cells were harvested and standardized. Samples were analyzed on 10% polyacrylamide gels containing 1 mg/ml gelatin type B (Sigma−Aldrich Corporation, San Luis, Mosby, MO, United States). After electrophoresis, gels were washed at room temperature for 1 h to remove sodium dodecyl sulfate (SDS) and incubated for 48 h at 37°C. Finally, gels were stained for 30 min with 0.25% Coomassie Brilliant Blue R−250 (Sigma-Aldrich Corporation, San Luis, Mosby, MO, United States) and destained in 10% (v/v) methanol and 5% (v/v) glacial acetic acid and were incubated overnight at 4°C. Degradation of the gelatin substrate by gelatinases was detected as white bands on a dark background. Bands were analyzed by the ImageJ 1.51r^®^ software package (Bethesda, MD, United States).

The presence of gelatinolytic bands of matrix metalloproteinase-2 (MMP-2) was determined according to the Rf of the run and comparing them with commercial standards of prestained molecular weight (range: 250–25 kDa). The latent forms of pro-MMPs were visualized in the gels due to the presence of SDS that destabilizes the binding of cysteine to the zinc atom, which keeps the zymogen inactive. The conditioned medium of the HTR-8/SVneo cell line was used as a positive control.

### Invasion Assay

Extravillous trophoblast cell invasion was assayed using a transwell insert (Millicell^®^, EMD Millipore Corporation, Darmstadt, Germany, United Kingdom). Swan 71 cells were plated (5 × 10^4^ cells/well) in the upper layer of transwell inserts (6.5 mm diameter polycarbonate membrane, 8 μm pore) precoated with 50 μl of extracellular matrix (ECM) (0.3 mg/ml) (Sigma-Aldrich Corporation, San Luis, Mosby, MO, United States) and cultured in complete DMEM/F-12 supplemented with 0.5% FBS with or without the sucrose solution, while 500 μl of complete DMEM/F-12 supplemented with 10% FBS was added to the lower chamber. After 24 h, the non-invading cells were removed from the upper surface of each chamber by a cotton swab and transwell membranes were fixed with methanol and stained with H&E. The number of invaded cells/field was quantified using the ImageJ 1.51r^®^ software package (Bethesda, MD, United States).

### Tube-Like Formation Assay

According to previously described methods (Reppetti et al., 2020), ECM gel reduced in growth factors (Sigma-Aldrich Corporation, San Luis, Mosby, MO, United States) was coated in a 96−well plate (50 μl) at 37°C, 5% CO_2_ for 30 min. After the treatments, Swan 71 cells were trypsinized and 100 μl of cells at 5 × 10^5^/ml were seeded onto the presolidified growth factor reduced ECM in the wells with 5% FBS in the medium. The tube-like formation was examined at 0, 4, and 6 h and photographed using an inverted microscope (Nikon, Eclipse E:200) equipped with a camera. Images were acquired from each well and quantified by the measurement of different morphological parameters using the plugin Angiogenesis Analyzer in the ImageJ 1.51r^®^ software package (Bethesda, MD, United States).

### Western Blot

As described previously (Reppetti et al., 2019), control cells and hyperosmolarity-treated cells were collected in a lysis buffer. Then, protein concentration was determined using the commercial Pierce BCA Protein Assay Kit (Thermo Fisher Scientific, Waltham, MA, United States).

For immunoblotting studies, 50 μg proteins from each sample of the lysate of Swan 71 cells were loaded and resolved on a 12.5% polyacrylamide gel and onto nitrocellulose membranes (Hybond ECL, Amersham Pharmacia Biotech Ltd., Pittsburgh, PA, United States). After blocking, membranes were rinsed and incubated overnight with an anti-Cav-1 (1:1,000; Santa Cruz Biotechnology Incorporation, CA, United States) followed by incubation with a peroxidase-conjugated secondary antibody (1:10,000; Jackson Immuno Research Laboratories, Incorporation, West Grove, PA, United States). Immunoreactivity was detected using the enhanced chemiluminescence (ECL) detection reagent (Amersham ECL plus, GE Healthcare Life Sciences, Pittsburgh, PA, United States). Bands were quantified by densitometric analysis using the ImageJ 1.51r^®^ software package (Bethesda, MD, United States). Results were expressed as the relative abundance of each protein and we used Ponceau staining as a loading control (Gilda and Gomes, 2013).

### Transmission Electron Microscopy

Swan 71 control cells and hyperosmolarity-treated cells were removed from culture plates with a cell scraper, washed with phosphate buffer or PBS 0.1 M, pH 7.4, and were fixed in the glutaraldehyde fixation solution 2.5% in phosphate buffer 0.1 M, pH 7.4 for 4 h at 4°C. Then, cells were washed twice with phosphate buffer or PBS 0.1 M, pH 7.4, and again fixed in 1% osmium tetroxide in 0.1 M phosphate buffer for 60 min at 4°C.

The material was then washed twice with bidistilled H_2_O and was dehydrated in ascending alcohols (50, 70, 96, 100°), two changes of each for 15 min, and, finally, two changes of acetone for 10 min. The material was included in “Durcupan” epoxy resin, which polymerized at 60°C for 72 h. Once polymerized, 0.5 μm semi-fine cuts were made using an ultramicrotome (Reichert–Jung Ultracut E) with a glass knife. The sections were mounted on slides and stained with toluidine blue for observation under the light microscope.

To observe the samples under the transmission electron microscope, ultrathin sections of 70–90 nm thick were made in the same ultramicrotome, the sections were mounted on copper grids, and were contrasted with uranyl acetate and lead citrate (Reynolds method). Samples were observed under a transmission electron microscope the MET Zeiss 109 equipped with a digital camera Gatan 1,000 W.

### Statistical Analysis

The statistical analysis of data was performed by the GraphPad Prism version 5 software (GraphPad Software Incorporation, La Jolla, CA, United States). All the values were expressed as mean ± SEM. Experiments were independently conducted in triplicate. The significance of the results was analyzed by the Student’s *t*-test for the comparison of two samples or by the one-way ANOVA followed by the Bonferroni *post-hoc* tests. Differences were considered significant at *p* < 0.05.

## Results

### Effect of Hyperosmolarity on Swan 71 Cell Viability

**FIGURE 1:**
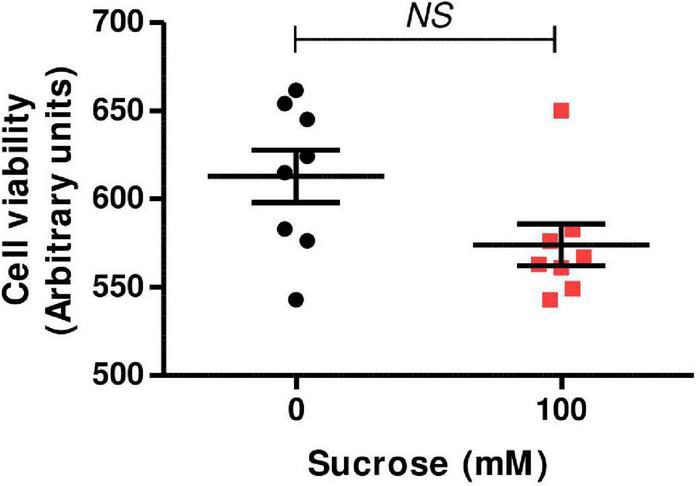
Effect of hyperosmolarity on Swan 71 cell viability. Swan 71 cells were cultured in iso-osmolarity (0 mM of sucrose—control cells) and hyperosmolarity (100 mM of sucrose—hyperosmolarity-treated cells) for 24 h and 3-(4,5-dimethylthiazol-2-yl)-2,5-diphenyl tetrazolium bromide (MTT) assay was performed. No significant differences were found in cell viability between the control and the hyperosmolar condition (*NS*: non-significant, *n* = 8 independent experiments). Values are plotted for each experiment and mean ± SEM is indicated. The Student’s *t*-test was applied for comparisons between the two groups.

Swan 71 cells were cultured in iso-osmolar condition (0 mM of sucrose—control cells) and hyperosmolar condition (100 mM of sucrose—hyperosmolarity-treated cells) for 24 h and cell viability was analyzed by MTT incorporation, a measure for mitochondrial dehydrogenase enzymatic activity. We observed that the addition of 100 mM of sucrose did not modify cell viability compared to control (*p* > 0.05, *n* = 8, Figure 1).

### Effect of Hyperosmolarity on Swan 71 Cell Migration

In order to study the effect of hyperosmolarity on the migration of EVT cells, we cultured Swan 71 cells in iso-osmolar and hyperosmolar conditions. We performed the “*in vitro* scratch test” to study cell migration and to evaluate the effect of sucrose on the repair of wounds produced in Swan 71 cell monolayers.

We calculate the percentage of wound healing and the migration speed.

**FIGURE 2:**
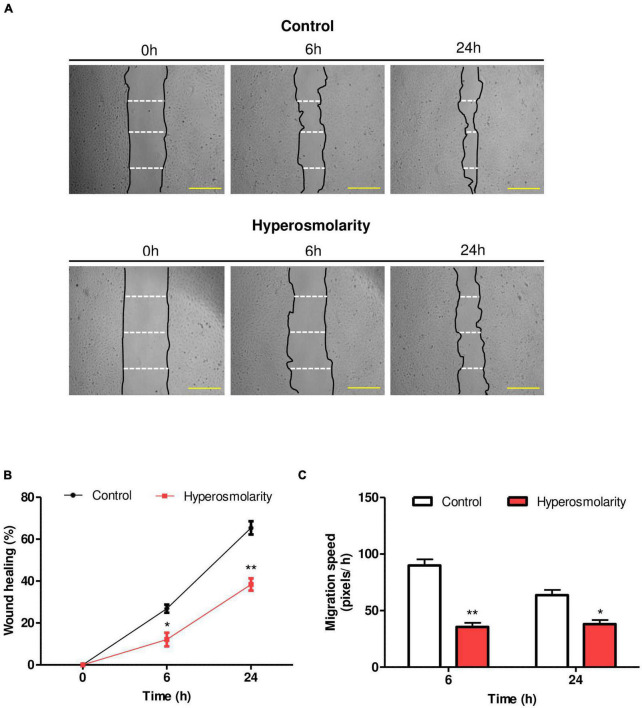
Effect of hyperosmolarity on Swan 71 cell migration. **(A)** Representative images of the wound made on the monolayer of Swan 71 cells at 0, 6, and 24 h treated with iso-osmolarity (control) or hyperosmolarity. Scale bar: 50 μm. **(B)** The ImageJ 1.51r^®^ software package was used to quantify the wound area and the results were expressed as wound healing percentage compared with time 0 h. The wound healing percentage was significantly lower in hyperosmolarity-treated cells compared to control cells at 6 and 24 h Values are presented as mean ± SEM (*p < 0.05, **p < 0.01, *n* = 3 independent experiments). ANOVA followed by the Bonferroni *post–hoc* test was applied. **(C)** The ImageJ 1.51r^®^ software package was used to quantify the migration speed of the cells. In hyperosmolarity, the migration speed decreased significantly compared to the control at 6 and 24 h. Values are presented as mean ± SEM (*p < 0.05, **p < 0.01, *n* = 3 independent experiments). ANOVA followed by the Bonferroni *post–hoc* test was applied.

We found that hyperosmolarity impaired the repairing process (Figure 2A). With respect to the percentage of wound healing, we found that it was significantly lower in hyperosmolarity-treated cells compared to control cells at 6 h: 12.11 ± 3.25 vs. 26.83 ± 0.94% (*p* < 0.05, *n* = 3) and at 24 h: 38.37 ± 2.91 vs. 65.38 ± 3.17% (*p* < 0.01, *n* = 3, Figure 2B). Concerning migration speed, we found that in the hyperosmolar condition it decreased significantly compared to the control at 6 h: 35.61 ± 3.63 vs. 89.93 ± 5.39 pixels/h (*p* < 0.01, *n* = 3) and 24 h: 38.08 ± 3.60 vs. 63.74 ± 4.58 pixels/h (*p* < 0.05, *n* = 3), being more important at 6 h (Figure 2C).

### Effect of Hyperosmolarity on Swan 71 Cells Metalloproteinases Activity

The invasion of the EVT cells into the decidua requires the action of proteases to degrade components of the ECM (including collagen and proteoglycan) (Nagaset and Woessner, 1999). We studied the gelatinolytic activity of MMPs by gelatin zymography of supernatants collected from Swan 71 control cells or hyperosmolarity-treated cells.

**FIGURE 3:**
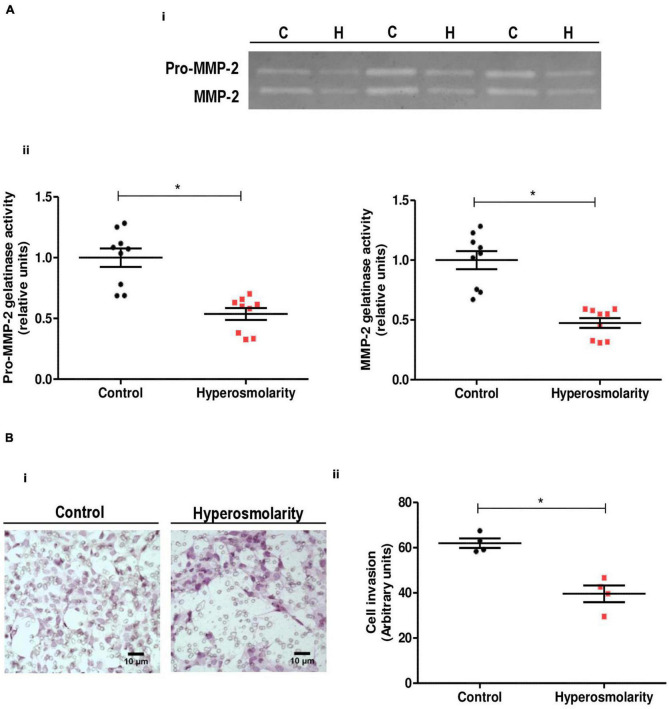
Effect of hyperosmolarity on Swan 71 cells matrix metalloproteinases (MMPs) activity and cell invasion through extracellular matrix (ECM)–coated transwells. **(A)** (I) Representative gelatin zymography of culture media from control (C) or hyperosmolarity–treated (H) Swan 71 cells. (II) The relative abundance of pro-MMP–2 and MMP-2 activity was performed by densitometric analysis of the gelatin zymography using the ImageJ 1.51r^®^ software package. Hyperosmolarity decreased both the pro-MMP–2 and MMP-2 activity compared to control (*p < 0.05, *n* = 3 independent experiments). Values are plotted for each experiment and mean ± SEM is indicated. The Student’s *t*-test was applied for comparisons between the two groups. **(B)** (I) Representative images of Swan 71 cells seeded on transwell membranes coated with ECM. Cells were cultured in iso-osmolarity or hyperosmolarity and photographs were taken after 24 h. Cells were stained with H&E (*magnification 400X*). (II) The quantification of the cell invasion was performed using the ImageJ 1.51r^®^ software package. The hyperosmolar treatment decreased the extravillous trophoblast (EVT) cells invasion of the reconstituted ECM compared to control after 24 h (*p < 0.05, *n* = 4 independent experiments). The mean of each independent experiment was plotted and the Student’s *t*-test was applied for comparisons between the two groups.

Supernatants obtained from HTR-8/SVneo cells were used as positive controls for MMP-2 activity (data not shown). MMP-9 latent/active forms were not detected in Swan 71 cells under our experimental conditions. We found that hyperosmolarity decreased both the pro-MMP-2 and MMP-2 activity compared to control (*p* < 0.05, *n* = 3, Figure 3A).

### Effect of Hyperosmolarity on Swan 71 Cell Invasion

We assessed the effect of hyperosmolarity on Swan 71 cell invasion in an ECM-based invasion assay. In this assay, invading cells found in the transwell membrane were stained with H&E to facilitate their identification and allow their quantification. Accordingly to the MMPs activity results, we found that hyperosmolarity decreased the Swan 71 cells invasion of the reconstituted ECM compared to control after 24 h (*p* < 0.05, *n* = 4, Figure 3B).

### Effect of Hyperosmolarity on the Endovascular Differentiation of Swan 71 Cells

**FIGURE 4:**
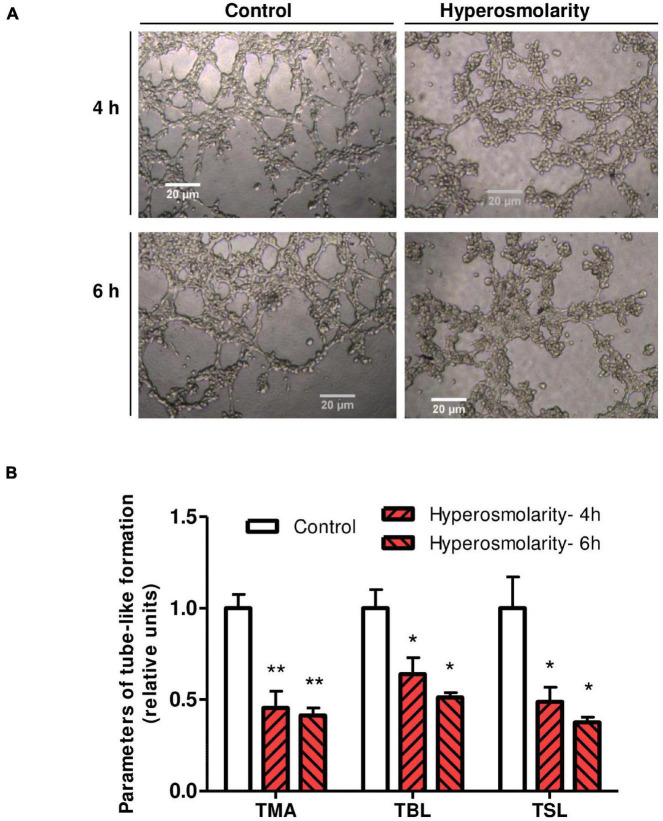
Effect of hyperosmolarity on endovascular differentiation of Swan 71 cells. **(A)** Representative images of tube–like formation at 4 and 6 h in Swan 71 cells after iso-osmolarity (control) or hyperosmolarity treatment (magnification 100X). **(B)** The ImageJ 1.51r^®^ software package with the Angiogenesis Analyzer plugin was used to analyze different parameters of Swan 71 tube–like formation. All the analyzed parameters decreased significantly after culturing cells in hyperosmolarity compared to control. Values are presented as mean ± SEM (**p < 0.01, *p < 0.05, *n* = 4 independent experiments). ANOVA followed by the Bonferroni *post–hoc* test was applied. TMA, total mesh area; TBL, total branching length; TSL, total segment length.

To explore the angiogenic response of Swan 71 cells after the hyperosmolar treatment, a tube-like formation assay was performed. We found that hyperosmolarity impaired the tube-like formation process at 4 and 6 h measured by the total meshes area (*p* < 0.01, *n* = 4), the total branching length (*p* < 0.05, *n* = 4), and the total segment length (*p* < 0.05, *n* = 4) compared to control (Figure 4).

### Effect of Hyperosmolarity on Cav-1 Protein Abundance in Swan 71 Cells

**FIGURE 5:**
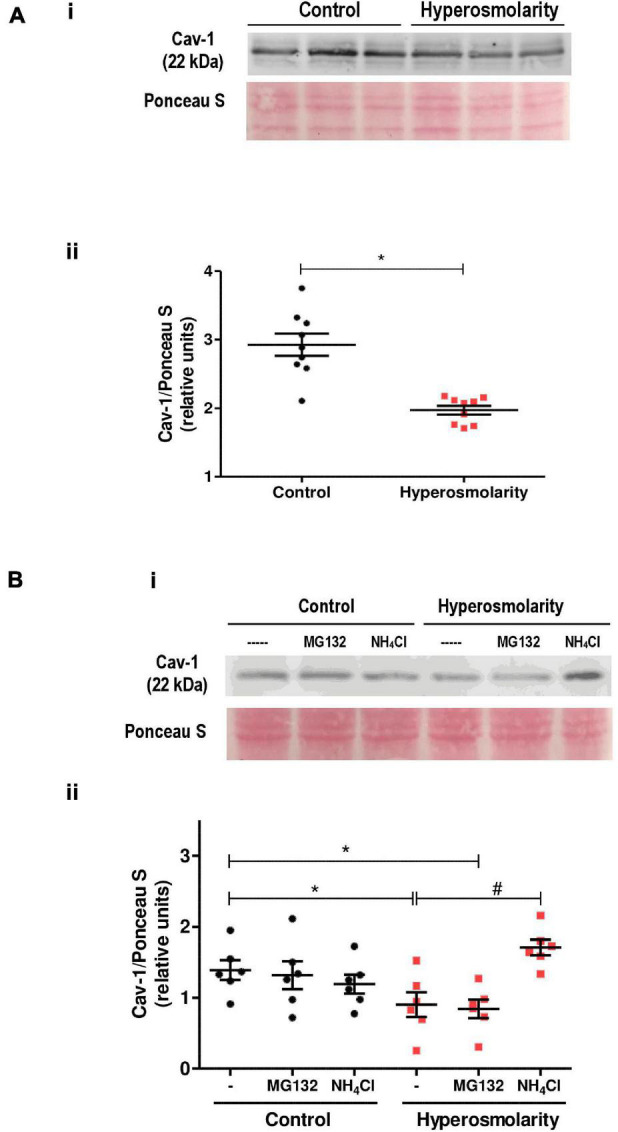
Effect of hyperosmolarity on caveolin-1 (Cav-1) expression in Swan 71 cells. **(A)** (I) Representative western blot of Cav-1 in Swan 71 cells grown in control (C: 0 mM of sucrose) or hyperosmolarity (H: 100 mM of sucrose). (II) The relative abundance of Cav-1 was determined by densitometric analysis of the western blot using the ImageJ 1.51r^®^ software package. The analysis showed an expected band of 22 kDa corresponding to Cav-1. However, the relative abundance of Cav-1 protein decreased significantly after cells were cultured in hyperosmolarity compared to control (*p < 0.05 vs. control, *n* = 3 independent experiments). Values are plotted for each experiment and mean ± SEM is indicated. The Student’s *t*-test was applied for comparisons between the two groups. **(B)** (I) Representative western blot of Cav-1 in Swan 71 cells cultured in control or hyperosmolarity and treated for 6 h with MG132 or ammonium chloride (NH_4_Cl). (II) The analysis of the western blot showed an expected band of 22 kDa corresponding to Cav-1. No significant differences were found in the relative abundance of Cav-1 among the controls [iso-osmolar medium supplemented with MG132, NH_4_Cl, or phosphate-buffered saline (PBS) used as a vehicle]. The addition of MG132 to block the proteasomal activity did not prevent Cav-1 degradation when cells were cultured in the hyperosmolar condition. However, the addition of NH_4_Cl to inhibit the lysosomal pathway avoided the Cav-1 degradation. (*p < 0.05 vs. control, #*p* < 0.01 vs. hyperosmolarity, *n* = 3 independent experiments). Values are plotted for each experiment and mean ± SEM is indicated. ANOVA followed by the Bonferroni *post–hoc* test was applied.

Caveolin-1 expression was explored in Swan 71 cells cultured in iso-osmolarity and hyperosmolarity. Western blot analysis showed an expected band of 22 kDa corresponding to Cav-1. However, the relative abundance of Cav-1 protein significantly decreased after the hyperosmolar treatment compared to control (*p* < 0.05, *n* = 3, Figure 5A).

In subsequent experiments, we assessed whether the proteasome-dependent proteolytic pathway or the lysosome degradation pathway is involved in the reduced expression of Cav-1 protein.

Swan 71 cells were cultured under iso-osmolar and hyperosmolar conditions and were treated for 6 h with MG132, a 26S proteasome inhibitor, or the lysosomal inhibitor NH_4_Cl.

The addition of the inhibitors to the control cells did not modify Cav-1 levels nor did the addition of the proteasome inhibitor MG132 to hyperosmolar-treated cells. Nevertheless, when the hyperosmolar-treated cells were incubated in presence of NH_4_Cl, we did not observe a decrease in Cav-1 levels previously found in hyperosmolarity, indicating that NH_4_Cl prevented the Cav-1 protein degradation in hyperosmolarity (*p* < 0.01 vs. hyperosmolarity, *n* = 3, Figure 5B).

### Effect of Hyperosmolarity on the Ultrastructure of Swan71 Cells

**FIGURE 6:**
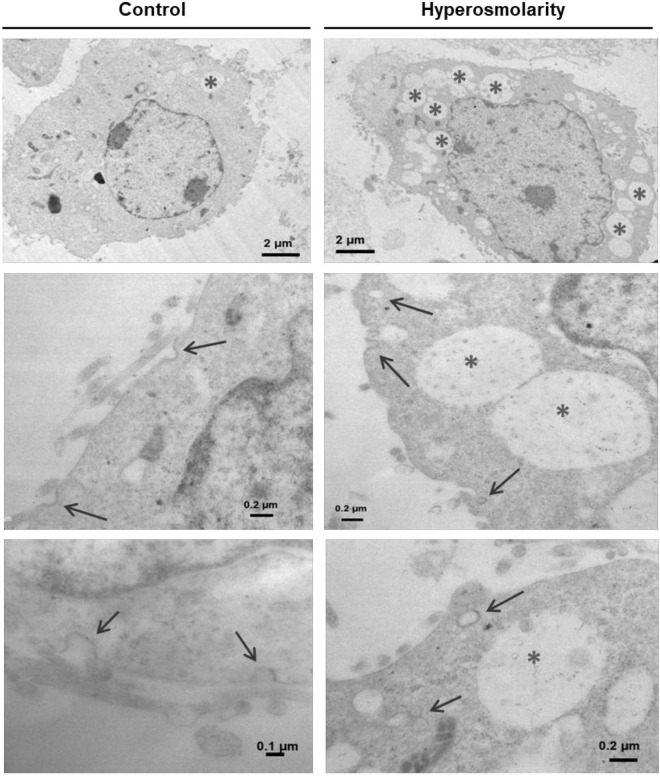
Effect of hyperosmolarity on the ultrastructure of Swan 71 cells. A transmission electron microscope was used to observe the ultrastructural changes in Swan 71 trophoblast cells cultured in iso-osmolarity (control) or hyperosmolarity. A greater number of vacuoles were found in the cytoplasm of cells cultured in hyperosmolarity compared to the control (asterisks). In addition, hyperosmolarity produced the internalization of structures compatible with caveolae (arrows).

Finally, we analyzed the ultrastructure of Swan 71 cells exposed to hyperosmotic stress by transmission electron microscopy (TEM). TEM images showed that hyperosmolarity produced the internalization of structures compatible with caveolae and a greater number of cytoplasmic vacuoles compared to controls (Figure 6).

## Discussion

During placentation, the EVT must correctly migrate and invade the maternal decidua and myometrium. Villous CTBs undergo a partial epithelial–mesenchymal transition (EMT) when differentiating into extravillous CTBs and gain the capacity to migrate and invade (Davies et al., 2016). The EVT cells acquire an endovascular phenotype and remodel maternal vessels. Dysregulation in the EVT differentiation processes results in several pregnancy complications as PE and FGR.

During pregnancy, trophoblast cells are very sensitive to minimal changes in osmolarity and, in some situations, hyperosmolarity may act as a lethal stressor (Liu et al., 2009; Pribenszky et al., 2010). In this regard, we have recently reported that osmolarities over 450 mOsM induce cell death by a mechanism that involves the activation of the TRPV-1 channel in EVT cells (Reppetti et al., 2019). In this study, we studied the effect of sublethal hyperosmolarity on the events related to endovascular-EVT differentiation.

Due to the difficulties in studying human EVT cells *in vivo*, we adopted an *in vitro* approach to investigate the effect of hyperosmolarity on the processes carried out by the EVT cells such as cell migration, invasion, and formation of tube-like structures, as a measure of endovascular differentiation.

We employed the Swan 71 cell line as a model of EVT, which previously demonstrated that spontaneously acquires an endovascular phenotype that forms tube-like structures when cells were cultured on an ECM (Reppetti et al., 2020). Other authors also observed this ability in EVT cells isolated from first trimester placenta (Dokras et al., 2001) and in immortalized cell lines such as HTR8/SVneo (Highet et al., 2012). Damsky and coworkers demonstrated that this phenomenon is regulated by the presence of laminin and type IV collagen in the Matrigel™ (Damsky et al., 1994) and reflects placental angiogenesis, endovascular differentiation, and acquisition of endothelial phenotype by the trophoblast (Highet et al., 2016).

We observed that Swan 71 cell migration and invasion decreased when cells were cultured in hyperosmolarity. In cell migration, we performed a spatiotemporal quantification. The EVT speed migration in the hyperosmolar condition was lower than in the control. The values of the cell velocity were consistent with the observed in the wound healing area. Our results are in agreement with previous studies describing that osmotic stress causes a significant decrease in cell migration and the migration speed of several cell types (Miermont et al., 2019). Nevertheless, in EVT cells, this alteration could have greater implications, since the success of trophoblast differentiation depends on the high migration capacity of these cells during the early stage of pregnancy. On the other hand, the EVT cell invasion into the decidua requires the secretion and activation of proteases to degrade the ECM. Here, we also found that both the latent and active forms of MMP-2 secreted by Swan 71 cells were reduced in hyperosmolarity. The MMPs have been involved in a wide range of physiologic and pathological remodeling processes. They have a critical role in tissue remodeling during development, wound healing, and also in the pathogenesis of several diseases (Sorokin, 2010). Both an increase and a reduction in the activity of MMPs may disturb physiological processes. As it was observed in human peritoneal mesothelial cells, hyperosmolarity markedly decreased MMP activity (Rougier et al., 1997). In trophoblast cells, this decrease in MMP activity can result in a superficial invasion of the decidua and the uterine spiral arteries by the trophoblast, impairing vascular remodeling and consequently reducing placental perfusion.

Finally, when we evaluated the ability of Swan 71 cells to differentiate into enEVT, we noted that hyperosmolarity significantly decreased the formation of tube-like structures.

In this study, we observed that sublethal hyperosmolarity produces a decrease in the cellular processes related to endovascular-EVT differentiation such as cell migration, cell invasion, and the formation of capillarity-like structures.

Alterations in the trophoblast differentiation processes were found in placentas from women who develop PE, leading to failures in the uterine spiral arteries remodeling (Zhou et al., 1997).

Previously, we reported that the disassembly of caveolae present in the membranes of EVT cells impaired the migration and endovascular differentiation of these cells (Reppetti et al., 2020).

Emerging data support the hypothesis that hyperosmolarity induces the internalization of the caveolae into the cytoplasm. In this study, it was described that the exposure of cardiomyocytes and fibroblasts to hyperosmolarity induces the caveola closed off from the plasma membrane and its internalization into the cytosol (Page et al., 1998; Kang et al., 2000).

It is well established that caveolae are stable structures. Thus, their turnover is slow and occurs by endocytosis and recycling mechanisms, maintaining a constant density of caveolae in the cell membrane (Pelkmans et al., 2004; Tagawa et al., 2005). Hence, under physiological conditions, Cav-1 is a long-lived protein. However, when caveolae stability is altered by different conditions, such as hyperosmolarity (Parton et al., 1994), Cav-1 turnover accelerates dramatically (Thomsen et al., 2002; Vassilieva et al., 2009; Hayer et al., 2010).

In addition, caveolae and Cav-1 are related to vesicular trafficking and signal transduction. Many signaling pathways that are involved in the regulation of trophoblast differentiation are located in caveolae (Moreau et al., 2002; Daoud et al., 2006; Xie et al., 2011). Concerning to endovascular EVT differentiation, Hu and coworkers demonstrated that both the phosphoinositide-3-kinase (PI3K)/protein kinase B (PKB or Akt) and the p38 the mitogen-activated protein kinase (MAPK) pathways are involved in the capillarity tube and network formation in EVT cells. In addition, the inhibition of these signaling routes led to a decrease in the formation of these tube-like structures. Interestingly, the receptors of these pathways are present in caveolae (Hu et al., 2010).

In this study, we evidenced that hyperosmolarity induced a decrease in the relative abundance of Cav-1 in Swan 71 cells. When we cultured these cells in hyperosmolarity in the presence of NH_4_Cl (that inhibits the lysosomal pathway by increasing the pH), we observed no degradation of Cav-1, indicating that this degradation pathway was activated in hyperosmolar condition and inhibited by NH_4_Cl. On the other hand, when Swan 71 cells were cultured in hyperosmolarity in the presence of the proteasome inhibitor MG132, we observed that there were no changes in the abundance of Cav-1 compared to Swan 71 cells cultured in hyperosmolarity, indicating that this degradation pathway was not activated in hyperosmolarity and it would not be involved in Cav-1 degradation in this condition. Therefore, we may infer that in the hyperosmolar condition, Cav-1 degradation in Swan 71 cells may occur by the lysosomal pathway.

As it was reported in other tissues (Page et al., 1998; Sinha et al., 2011), in EVTs cells, we also evidenced that the hyperosmolarity induced the internalization of caveolae.

Previous studies have shown that gestational pathologies associated with failures in the placentation such as hypertension in pregnancy, FGR, and PE have a hemoconcentration state with reduced plasma volumes, which results in hypertonic stress (Huisman and Aarnoudse, 1986; Mello et al., 2002).

In addition, we previously reported a reduced expression of Cav-1 in the trophoblast cells from preeclamptic placentas associated with alterations in the membrane lipid composition that impairs the normal caveolae formation and Cav-1 insertion in the plasma membrane (Levi et al., 2016).

Similar to what was reported in human corneal epithelial cells (Wang et al., 2019), we found a significant increase in the number of cytoplasmic vacuoles under the hyperosmolar condition, which may correspond to autophagic vacuoles where lysosomal proteolysis may be taking place.

**FIGURE 7:**
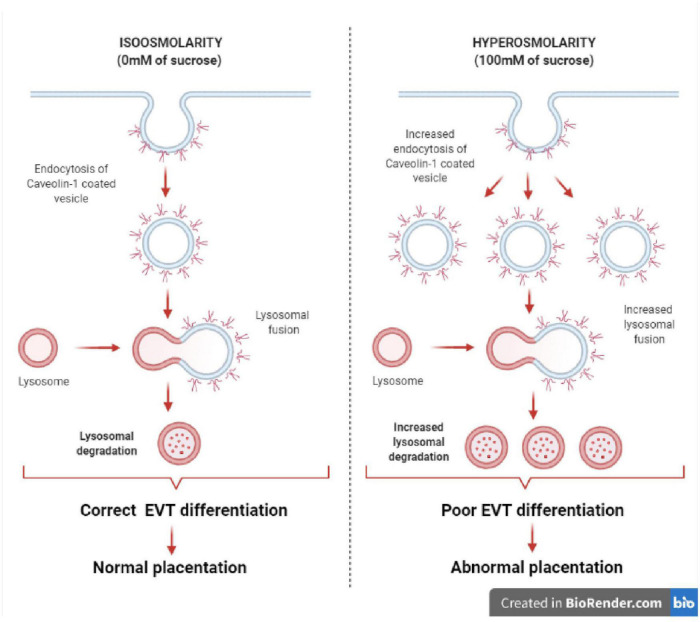
Effect of hyperosmolarity on Swan 71 cell differentiation. A graphical abstract was generated to illustrate the effect of hyperosmolarity on Swan 71 cell differentiation. Hyperosmolarity increases the internalization of caveolae in EVT cells, leading to a decrease in Cav-1 protein by lysosomal proteolysis. Therefore, signal transductions located in the caveolae and related to the modulation of EVT differentiation such as cell migration, invasion, and endovascular differentiation will be diminished and this could lead to abnormal placentation.

In conclusion, our findings show new evidence that sublethal hyperosmolarity may produce the internalization of the caveolae domains in EVT cells, leading to a decrease in Cav-1 protein by lysosomal proteolysis. Therefore, the disruption of this scaffolding platform, where are located many signaling pathways related to the modulation of EVT differentiation, may lead to abnormal placentation (Figure 7).

A better understanding of the molecular processes and signaling pathways located in the caveolae that regulate trophoblast differentiation in early gestation will improve our knowledge of the role of this platform in normal placental development. Therefore, alterations in the caveola structure induced by hyperosmolarity might result in pathologies associated with incomplete remodeling of the spiral arteries such as PE and FGR.

## Data Availability Statement

The raw data supporting the conclusions of this article will be made available by the authors, without undue reservation.

## Author Contributions

JR and YM carried out the experimental work. NM designed the study. JR carried out the analysis of data. JR, NM, and AD wrote and proofread the manuscript. MF, AD, and NM critically reviewed the manuscript. All authors contributed to the final version of the manuscript.

## Conflict of Interest

The authors declare that the research was conducted in the absence of any commercial or financial relationships that could be construed as a potential conflict of interest.

## Publisher’s Note

All claims expressed in this article are solely those of the authors and do not necessarily represent those of their affiliated organizations, or those of the publisher, the editors and the reviewers. Any product that may be evaluated in this article, or claim that may be made by its manufacturer, is not guaranteed or endorsed by the publisher.
